# Epidemiology of gastroenteropancreatic neuroendocrine neoplasms: a review and protocol presentation for bridging tumor registry data with the Italian association for neuroendocrine tumors (Itanet) national database

**DOI:** 10.1007/s12020-023-03649-4

**Published:** 2024-01-04

**Authors:** Francesco Panzuto, Stefano Partelli, Davide Campana, Filippo de Braud, Francesca Spada, Mauro Cives, Salvatore Tafuto, Alexia Bertuzzi, Fabio Gelsomino, Francesca Bergamo, Stefano Marcucci, Laura Mastrangelo, Sara Massironi, Marialuisa Appetecchia, Angelina Filice, Giuseppe Badalamenti, Mirco Bartolomei, Vito Amoroso, Luca Landoni, Maria Grazia Rodriquenz, Monica Valente, Annamaria Colao, Andrea Isidori, Giuseppe Fanciulli, Roberto Bollina, Michele Ciola, Giovanni Butturini, Riccardo Marconcini, Emanuela Arvat, Saverio Cinieri, Rossana Berardi, Sergio Baldari, Ferdinando Riccardi, Chiara Spoto, Dario Giuffrida, Domenico Gattuso, Diego Ferone, Maria Rinzivillo, Emilio Bertani, Annibale Versari, Alessandro Zerbi, Giuseppe Lamberti, Eleonora Lauricella, Sara Pusceddu, Nicola Fazio, Elisabetta Dell’Unto, Marco Marini, Massimo Falconi

**Affiliations:** 1 Department of Medical-Surgical Sciences and Translational Medicine, Sapienza University of Rome, Digestive Disease Unit, ENETS Center of Excellence, Sant’Andrea University Hospital, Rome, Italy; 2 Pancreatic Surgery Unit, Pancreas Translational and Clinical Research Center, ENETS Center of Excellence, IRCCS San Raffaele Hospital, Vita-Salute San Raffaele University, Milan, Italy; 3 Department of Medical and Surgical Sciences (DIMEC), Alma Mater Studiorum - University of Bologna, Medical Oncology Unit, IRCCS Azienda Ospedaliero-Universitaria di Bologna, Bologna, Italy; 4 Department of Medical Oncology, Fondazione IRCCS Istituto Nazionale Dei Tumori Di Milano, ENETS Center of Excellence, Milan, Italy; 5 Gastrointestinal and Neuroendocrine Tumors Oncology Unit - ENETS Center of Excellence, European Institute of Oncology (IEO) - IRCCS, Milan, Italy; 6 Dipartimento Interdisciplinare di Medicina, Università di Bari “Aldo Moro”, Bari, Italy; 7 S.C. Sarcomi e Tumori Rari, Istituto Nazionale Tumori, I.R.C.C.S. - Fondazione “G. Pascale”, ENETS Center of Excellence, Napoli, Italy; 8 Sezione Sarcomi/NET e Oncologia del Giovane Adulto (AYA-Adolescent Young Adult) Humanitas Research Hospital-IRCCS Via Manzoni 56, 20089 Rozzano, Milan, Italy; 9 Department of Oncology and Hematology, Division of Oncology, University Hospital of Modena, Modena, Italy; 10 Oncology 1, Veneto Institute of Oncology - IRCCS, Padua, Italy; 11 Department of Surgery & Hepato-Biliary and Pancreatic Unit Santa Chiara Hospital, Azienda Provinciale per i Servizi Sanitari (APSS), Trento, Italy; 12 UO Chirurgia Generale e d’Urgenza IRCCS Azienda Ospedaliera Universitaria Sant’Orsola Malpighi c/o Ospedale Maggiore, Bologna, Italy; 13 Division of Gastroenterology, Fondazione IRCCS San Gerardo dei Tintori, and University of Milano-Bicocca, School of Medicine, Monza, Italy; 14 Oncological Endocrinology Unit, Regina Elena National Cancer Institutre - IFO IRCCS, Roma, Italy; 15 Servizio di Medicina Nucleare, Azienda USL-IRCCS di Reggio Emilia, Reggio Emilia, Italy; 16 Department of Surgical, Oncological and Oral Sciences, University of Palermo, Palermo, Italy; 17 Nuclear Medicine Unit, Azienda Ospedaliera Universitaria-Ferrara, Ferrara, Italy; 18 Medical Oncology Unit, Department of Medical & Surgical Specialties, Radiological Sciences & Public Health, University of Brescia at Spedali Civili Hospital, Brescia, Italy; 19 Department of General and Pancreatic Surgery, The Pancreas Institute, University of Verona Hospital Trust, ENETS Center of Excellence, Verona, Italy; 20 Oncology Unit - Ospedale IRCCS Casa Sollievo della Sofferenza - San Giovanni Rotondo, Foggia, Italy; 21 Center for Immuno-Oncology, Oncology Department, University Hospital of Siena, Siena, Italy; 22 Department of Endocrinology University of Naples, Azienda Ospedaliera Universitaria “Federico II”, ENETS CEnter of Excellence, Napoli, Italy; 23 Department of Experimental Medicine, Sapienza University of Rome, Rome, Italy; 24 Endocrine Oncology Program, Endocrine Unit, University Hospital (AOU) of Sassari, Sassari, Italy; 25 Presidio Ospedaliero di Rho, Milan, Italy; 26 Regional Hospital of Bolzano, Bolzano, Italy; 27 HPB Surgery, P. Pederzoli Hospital, Peschiera del Garda, Verona, Italy; 28 Medical Oncology Unit, Azienda Ospedaliero-Universitaria Pisana, Pisa, Italy; 29 Oncological Endocrinology Unit, Department of Medical Sciences University of Turin, Turin, Italy; 30 Oncology Unit, A. Perrino Hospital, Brindisi, Italy; 31 Department of Medical Oncology, Università Politecnica Delle Marche, AOU Ospedali Riuniti Delle Marche, Ancona, Italy; 32 Nuclear Medicine Unit, Department of Biomedical and Dental Sciences and Morpho-Functional Imaging, University of Messina, Messina, Italy; 33 Medical Oncology Unit, Ospedale Cardarelli, Naples, Italy; 34 Medical Oncology, Santa Maria Goretti Hospital, Latina, Italy; 35 Medical Oncology Department, Istituto Oncologico del Mediterraneo, Viagrande, Catania, Italy; 36 Oncology Unit, S. Filippo Neri Hospital ASL Roma 1, Rome, Italy; 37 Endocrinology Unit, Department of Internal Medicine & Medical Specialties (DiMI), IRCCS Ospedale Policlinico San Martino, University of Genoa, Genoa, Italy; 38 Digestive Surgery, European Institute of Oncology IRCCS, ENETS Center of Excellence, Milan, Italy; 39 Humanitas Research Hospital –IRCCS, Pancreatic Surgery Unit, Rozzano, and Humanitas University, Department of Biomedical Sciences, Pieve Emanuele, Italy; 40 IMT School for Advanced Studies Lucca, Lucca, Italy

**Keywords:** Neuroendocrine tumors, epidemiology, registry, management, database.

## Abstract

**Abstract:**

Neuroendocrine neoplasms (NENs) are rare tumors with diverse clinical behaviors. Large databases like the Surveillance, Epidemiology, and End Results (SEER) program and national NEN registries have provided significant epidemiological knowledge, but they have limitations given the recent advancements in NEN diagnostics and treatments. For instance, newer imaging techniques and therapies have revolutionized NEN management, rendering older data less representative. Additionally, crucial parameters, like the Ki67 index, are missing from many databases. Acknowledging these gaps, the Italian Association for Neuroendocrine Tumors (Itanet) initiated a national multicenter prospective database in 2019, aiming to gather data on newly-diagnosed gastroenteropancreatic neuroendocrine (GEP) NENs. This observational study, coordinated by Itanet, includes patients from 37 Italian centers. The database, which is rigorously maintained and updated, focuses on diverse parameters including age, diagnostic techniques, tumor stage, treatments, and survival metrics. As of October 2023, data from 1,600 patients have been recorded, with an anticipation of reaching 3600 by the end of 2025. This study aims at understanding the epidemiology, clinical attributes, and treatment strategies for GEP-NENs in Italy, and to introduce the Itanet database project. Once comprehensive follow-up data will be acquired, the goal will be to discern predictors of treatment outcomes and disease prognosis. The Itanet database will offer an unparalleled, updated perspective on GEP-NENs, addressing the limitations of older databases and aiding in optimizing patient care.

**Study registration:**

This protocol was registered in clinicaltriasl.gov (NCT04282083).

## Introduction

Neuroendocrine neoplasms (NENs) are rare tumors with varied clinical behaviors. Their rising incidence, possibly linked to better diagnostic methods, increased awareness, or actual epidemiological changes, is noteworthy [1]. However, Europe’s epidemiological landscape of NENs remains inconsistent, influenced by diverse healthcare systems, recording practices, and genetic and environmental factors [2]. A key challenge in analyzing NEN epidemiology is the reliance on national tumor registries or long-term retrospective data collection. In Italy, the epidemiological data on NENs is sourced from the national report on rare cancers published in 2015 by the Italian Association of Tumor Registry [3]. This report indicated an overall incidence for GEP-NENs of approximately 2 per 100,000 individuals. The report unexpectedly showed a higher proportion of poorly differentiated carcinomas compared to well-differentiated tumors, contradicting data from many referral centers and highlighting limitations in national tumor registries’ data collection methods.

The divergence in data points to the need for a more robust, systematic approach to collecting epidemiological information on NENs that can accurately reflect the incidence and characteristics of these tumors in Italy. To address this topic, the Italian Association for Neuroendocrine Tumours (Itanet) launched a national multicenter prospective database in 2019 to capture data on newly diagnosed GEP NENs patients.

In the present study, a brief review of the major available epidemiological data on GEP-NENs is provided, with the aim of understanding the potential discrepancies among the different data sources. In addition, the Itanet study protocol is reported, and the expected findings are highlighted.

## The SEER program and other national tumor registries

Most epidemiological knowledge on GEP-NENs stems from national tumor registries, notably the SEER program in the US, which has been a major source of information [1]. These databases are crucial but have limitations in the dynamic field of NENs [4]. SEER’s comprehensive patient data is instrumental in understanding GEP-NEN incidence and prevalence and in developing survival prediction models [5]. However, it is worthy of noting that its data from 1975 to 2015 covers a period of considerable NEN diagnostic and treatment advancement. Changes in NEN classifications and coding over time may also influence the study’s findings. Innovative imaging including 68Ga-DOTATOC PET-CT and novel radiological and endoscopic techniques are not universally incorporated in the SEER database. Furthermore, the availability of “novel” treatments (i.e., targeted agents and radioligand therapy with 177Lu-Dotatate) has changed the therapeutic landscape of NENs [6]. Finally, the lack of crucial data in the SEER registries, like the Ki67—considered the main prognostic indicator for NENs [7]—challenges the analysis of SEER registry data and undermines its accuracy.

The recently published Bavarian registry, which included 9,236 cases recorded using the ICD-10 coding system, presents a methodological approach similar to that of other national tumor registries [8]. In this case too, due to the data collection methodology, some crucial information was missing, such as staging which was unavailable in 44.5% of the cases. In the study, only “malignant tumors” were included, excluding indolent NENs. This poses a significant confounding factor since, per WHO definition [9], all NENs can have malignant potential regardless of their behavior.

Recent data from the Japanese National Cancer Registry, initiated in 2016, reported on 6,735 cases, covering incidence, prevalence, and tumor characteristics [10]. While pioneering in Japanese epidemiological data, it faces common retrospective study limitations, including potential underestimation of NEN incidence by excluding non-‘malignant’ tumors, lacking data on key disease features, and limited generalizability outside Japan.

Norwegian Cancer Registry data on 17,128 NEN patients diagnosed between 1993 and 2015 were analyzed [11], using ICD-O 2nd and 3rd editions for coding and excluding post-mortem diagnoses or cases with zero follow-up. Tumor grading was unfeasible due to missing Ki67 and WHO grade data, leading to classifications based on morphology and behavior into low-intermediate and high aggressiveness. This approach introduces a bias in prognosis evaluation, typically reliant on WHO grading.

## National databases

Providing a broader perspective is the European Neuroendocrine Tumour Society (ENETS) registry. This initiative aggregated data from seven national NEN registries, comprising a substantial cohort of 10,102 patients from various regions in Europe [12]. The ENETS study included a combination of retrospective and prospective data, which were collected through the collaborative efforts of multiple national groups. This methodology introduces the possibility of selection biases, alongside other inherent limitations. Moreover, the research was subject to supplementary limitations, including the protracted timeframe during which data were gathered, encompassing instances preceding the year 2003. A considerable proportion of cases exhibited the absence of significant prognostic parameters. In approximately 1/3 of the cases, there was an absence of grading information, which presents difficulties in interpreting the study’s findings within the current context of neuroendocrine neoplasms. It is also worth noting that this initiative ended up in 2020.

A study from Spain, analyzing 907 tumors through a National Cancer Registry, revealed valuable insights into the epidemiology, clinical practices, and prognosis of GEP NENs [13]. However, it is again important to note that the data from the Spanish registry were collected from 2008 to 2011, prior to the introduction of more recent diagnostic and therapeutic modalities (i.e., targeted agents and radioligand therapy with 177Lu-Dotatate). Consequently, the figures derived from these data might not reflect the current scenario.

A comprehensive population-based cohort study was conducted in the UK, involving all patients diagnosed with NENs between 2012 and 2015, as recorded in the national registry using the ICD-10 coding system [14]. This study encompassed 15,145 patients, approximately half of whom had GEP-NENs. It reported on tumor features, disease staging, incidence, and overall survival. However, due to the nature of the study’s reliance on tumor registry data, significant information was missing; for example, staging was available for only 61% of cases. Additionally, a number of indolent tumors, which were categorized as ‘benign,’ were not included in the registry.

A similar epidemiological study was reported in Switzerland [15], where all NEN diagnoses were collected from the national registry spanning from 1976 to 2016. However, the extremely long duration of the study and the lack of crucial data (e.g., tumor grade was available in only 24% of cases) affected the study’s robustness for reasons similar to those mentioned above.

In 2010, a prospective study was conducted in Austria to assess GEP-NETs, aiming to determine their incidence and main clinical and pathological characteristics. However, this study only collected data from 285 patients over a one-year period, limiting its ability to offer insights into therapeutic approaches and long-term follow-up [16].

## The Itanet database project

The primary objective of the Itanet database is to understand the epidemiology, presentation modalities, clinical and pathological features, and diagnostic-therapeutic approaches employed by participating centers when managing a GEP-NEN patient in Italy. Additionally, once adequate follow-up data becomes available, it aims at identifying predictors of treatment response and determine prognostic factors for disease recurrence/progression as well as overall survival. This is a multicenter prospective observational study coordinated by Itanet, including consecutive patients newly diagnosed with GEP-NENs. The study involves 37 Italian centers (supplementary file), including all the Italian ENETS Centers of Excellence, that agreed to participate following an invitation from Itanet. Prior to initiating enrollment, the study was registered on ClinicalTrials.gov (NCT04282083). Each participating center obtained approval from its Ethics Committee, and full written informed consent was obtained from each patient before their data inclusion in the database. A web-based CRF (https://itanetdb.fullcro.org/login.aspx) was utilized for data collection, where pseudo-anonymized data with unique patient identifiers were inputted.

The database includes adult patients with diagnosis of GEP-NEN confirmed either by histology/cytology or supported by positive 68Ga-DOTATOC PET-CT findings along with at least one secondary level contrastographic imaging (either CT scan or MRI), made within 1 year prior to signing the informed consent.

**Fig. 1:**
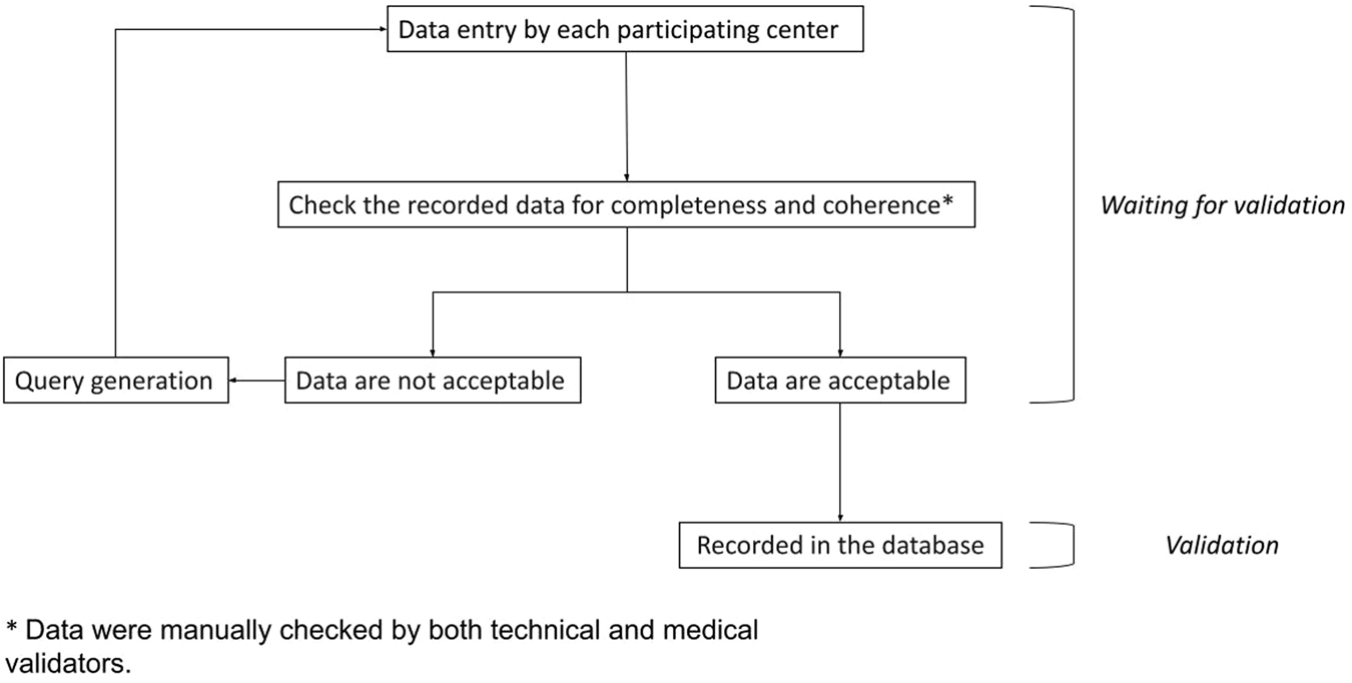
The figure illustrates the flow of patient data included in the database. Data are initially collected and then subjected to manual double-checking by both technical and medical validators prior to definitive storage. Should any discrepancies or missing information arise, the validators generate queries that are sent to the respective center for clarification, ensuring that only verified data are accepted as valid

The data validation process was conducted by two data managers with access to anonymized data. One was responsible for managing the CRF (designated as the “technical validator”), while the other was a physician from the Itanet research team (MR, designated as the “medical validator”). The validation was executed in two phases: in the initial phase, the technical validator manually inspected for missing data and assessed for any duplicate entries, which might arise if a patient was seen in more than one center within a short period following the initial diagnosis. In the second phase, the medical validator examined the data to ensure its accuracy and consistency. If discrepancies arose during the validation process, a query was generated and forwarded to the respective center, which was then requested to resolve them before the data was definitively stored in the database. Detailed data flow is reported in Fig. 1. The primary data extracted from medical records include age, gender, functional syndrome, familial hereditary syndrome, interval between symptom onset and NEN diagnosis, diagnostic procedures at the time of initial diagnosis, primary tumor location, histopathological features (in line with the WHO classification) [9], immunohistochemical characteristics, Ki67 value, tumor stage at diagnosis, therapeutic interventions, data on time and mode of tumor recurrence/progression, and survival. The database is updated after each follow-up visit, which is scheduled by each center based on clinical practice and the specific needs of the patient. The first patient’s data was logged in May 2019. As of October 2023, the database holds records for 1,600 patients. Currently, the enrollment rate is approximately 800 patients per year, and it is expected that the target number of 3,600 patients anticipated by the protocol will be reached by the end of 2025.

Key parameters will be presented using descriptive statistics. Where appropriate, the chi-square or Fisher’s exact test will be employed to compare variables. Continuous variables will be compared between groups using one-way analysis of variance. Overall survival will be calculated from the date of diagnosis to either the date of death from any cause or the last follow-up for patients still alive, using Kaplan–Meier curves. Differences among patient subgroups will be analyzed using log-rank tests. For the identification of independent prognostic factors, Cox proportional hazards multivariate analyses will be conducted.

## Discussion

Gaining a comprehensive epidemiological understanding of GEP-NENs is challenging due to the inherent heterogeneity and often incomplete nature of data derived from tumor registries or retrospective databases [1, 4].

These sources encompass extensive time periods during which diagnostic and therapeutic strategies for these tumors underwent significant evolution. In the past 15 years, the advent of 68Ga-DOTATOC PET-CT, coupled with advances in endoscopy and radiology, has transformed the diagnostic landscape for GEP-NENs. This led to a notable rise in incidentally discovered tumors and enhanced capabilities in disease staging [17]. Additionally, the possibilities for treatment have substantially changed, especially for well-differentiated G1-G2 tumors, owing to the registration of targeted drugs in 2011 and the approval of 177Lu-Dotatate for radioligand therapy in 2018. The WHO classification has also been recently updated with the introduction of the well-differentiated NET G3 category (well-differentiated tumors with Ki67 > 20%) [9]. Amidst these changes, the intrinsic heterogeneity of GEP-NENs persists, complicating accurate prognostic analyses without key information like Ki67 values and accurate disease staging. Hence, older tumor registries, which often overlook these nuances, are inadequate. There is an urgent demand for contemporary, comprehensive databases that can offer trustworthy insights into the clinical trajectory of GEP-NENs.

The Itanet national database aims at overcoming these limitations and addressing this significant gap in knowledge. The project has several potential notable strengths: (i) It follows a prospective study design; (ii) A data quality check is implemented (Fig. (1); (iii) it ensures completeness by prompting participating centers to input essential data into the e-CRF if not already done; (iv) It involves a comprehensive network of centers, well-distributed across the country, actively managing GEP-NEN patients; (v) Participants hail from a genuinely multidisciplinary background, representing expertise in areas like surgery, medical oncology, gastroenterology, endocrinology, and nuclear medicine.

Admittedly, this study has limitations. It does not encompass all GEP-NEN cases nationally and, thus, should be viewed more as a substantial database rather than a comprehensive national tumor registry. The multicenter nature, inherent to such studies, may introduce variability due to different diagnostic and therapeutic approaches across participating centers. Centralized histological or radiological reviews were not feasible due to the study’s structure and the extensive number of centers involved.

Nevertheless, we contend that these data can guide physicians treating GEP-NENs, shedding light on the incidence and presentation patterns of these tumors, and offering insights that may aid in determining the most effective therapeutic sequencing for these patients.

### Supplementary Information

Supplementary file 1
