# Supplementary material for: Epidemiology of gastroenteropancreatic neuroendocrine neoplasms: a review and protocol presentation for bridging tumor registry data with the Italian association for neuroendocrine tumors (Itanet) national database
Source: Endocrine. 2024 Jan 4;84(1):42–7. doi: 10.1007/s12020-023-03649-4 (PMC10987336; doi:10.1007/s12020-023-03649-4)
Supplement: Supplementary file 1 [file 12020_2023_3649_MOESM1_ESM.docx]

**Supplementary file: list of participating centers**

1. IRCCS Ospedale San Raffaele-Milano – ENETS Center of Excellence
2. Azienda Ospedaliera Universitaria Policlinico S. Orsola Malpighi-Bologna – ENETS Center of Excellence
3. IRCCS Istituto Nazionale Tumori-Milano – ENETS Center of Excellence
4. Azienda Ospedaliero-Universitaria S. Andrea-Roma – ENETS Center of Excellence
5. Istituto Europeo di Oncologia-Milano – ENETS Center of Excellence
6. Azienda Ospedaliera Universitaria Consorziale Policlinico-Bari
7. Istituto Tumori IRCCS Fondazione G. Pascale-Napoli – ENETS Center of Excellence
8. Istituto Clinico Humanitas-Rozzano – ENETS Center of Excellence
9. Azienda Ospedaliera Universitaria, Policlinico-Modena
10. DOFIP-Dipartimento Oncologico Funzionale Interaziendale di Padova
11. Ospedale Santa Chiara (APSS), Trento
12. Alma mater studiorum, IRCCS Azienda ospedaliera - universitaria di Bologna
13. Ospedale S. Gerardo-Monza
14. IRCCS Istituti Fisioterapici Ospitalieri-Roma
15. Azienda USL-IRCCS di Reggio Emilia
16. Centro di Riferimento Regionale per i Tumori Rari dell'adulto, Università degli studi di Palermo
17. Azienda Ospedaliera Universitaria-Ferrara
18. Spedali Civili-Brescia
19. Azienda Ospedaliera Universitaria integrata-Verona – ENETS Center of Excellence
20. Casa Sollievo della Sofferenza, S. Giovanni Rotondo
21. Ospedale Universitario di Siena
22. Policlinico Università Federico II-Napoli – ENETS Center of Excellence
23. Policlinico Umberto I-Roma
24. Azienda Ospedaliera Universitaria-Sassari
25. Presidio Ospedaliero di Rho-Rho
26. Ospedale regionale-Bolzano
27. Clinica Pederzoli-Peschiera del Garda (VR)
28. Ospedale Santa Chiara-Pisa
29. Azienda Ospedaliero Universitaria Città della salute e della Scienza –Torino
30. P.O. “A.Perrino” Brindisi
31. AOU Ospedali Riuniti_Ancona
32. Azienda Ospedaliera Universitaria Policlinico G. Martino-Messina
33. Azienda Ospedaliera di rilievo nazionale A. Cardarelli-Napoli – ENETS Center of Excellence
34. Ospedale S. Maria Goretti-Latina
35. Istituto Oncologico del Mediterraneo-Catania
36. Ospedale S. Filippo Neri_Roma_
37. Ospedale Policlinico S. Martino-Genova
